# Measuring habituation to stimuli: The Italian version of the Sensory Habituation Questionnaire

**DOI:** 10.1371/journal.pone.0309030

**Published:** 2024-12-31

**Authors:** Vincenza Tarantino, Noemi Passerello, Ayelet Ben-Sasson, Tamar Y. Podoly, Alessia Santostefano, Massimiliano Oliveri, Laura Mandolesi, Patrizia Turriziani

**Affiliations:** 1 Department of Psychology, Educational Sciences and Human Movement, University of Palermo, Palermo, Italy; 2 Department of Humanities, “Federico II” University, Naples, Italy; 3 Department of Occupational Therapy, Faculty of Social Welfare and Health Sciences, University of Haifa, Mount Carmel, Haifa, Israel; 4 School of Psychological Sciences, University of Haifa, Mount Carmel, Haifa, Israel; 5 International School of Advanced Studies, University of Camerino, Camerino, Italy; 6 Department of Biomedicine, Neurosciences and Advanced Diagnostics, University of Palermo, Palermo, Italy; Universita degli Studi di Udine, ITALY

## Abstract

Sensory habituation allows us to decrease responsiveness to repetitive or prolonged stimuli over time, making them easy to filter out and not interfere with ongoing activities. As such, habituation could be an important aspect to be evaluated within a sensory and cognitive assessment. The main aim of the present study was to validate an Italian version of the Sensory Habituation Questionnaire (S-Hab-Q), a self-report tool assessing how long an adult individual takes to adapt to daily sensory stimuli. We examined the relationship between sensory habituation and sensory sensitivity by administering the Sensory Perception Quotient questionnaire (SPQ) and tested a factor model based on a sensory modality categorization of items. In addition, given the high probability of altered sensory processing in autism, we explored the relationship between sensory habituation and autistic traits by administering the Autism Quotient questionnaire (AQ). A total of 262 participants, aged 18 to 67 years, completed the S-Hab-Q, the SPQ, and the AQ questionnaires. The results showed that, as the original version of the S-Hab-Q, the Italian version had a high internal consistency and a significant correlation with the SPQ score. A confirmatory factor analysis, based on a two-factor model (i.e., vision and hearing vs. touch, smell, and taste), showed a good fit of the S-Hab-Q data. As expected, a significant correlation between the S-Hab-Q and the AQ score was found. Interestingly, mediation analysis revealed that the S-Hab-Q score mediated the relationship between SPQ and AQ scores. Overall, the results confirm that a questionnaire assessing habituation can be a feasible tool to profile individual habituation in daily life. Moreover, they suggest that sensory habituation contributes to explaining the link between sensory sensitivity and autistic traits.

## Introduction

Individuals exhibit unique responses to the same sensory inputs, even under identical stimulus features (e.g., intensity, frequency, and duration). From a psychophysiological perspective, the response characteristics depend on several individual factors, such as the sensory threshold and habituation levels [1–4]. The sensory threshold refers to the minimum intensity of a stimulus required for detecting or reacting to it. Habituation, on the other hand, involves processes leading to a decrease in the response intensity to a stimulus with prolonged or repeated over time [5]. This phenomenon serves an evolutionary purpose, enabling humans and animals to ignore irrelevant information, even when the sensory threshold is low. For instance, the ability to filter out noise in the hallway, a colleague’s perfume, or the room’s temperature were crucial for effective revision of this paper.

The concept of sensory sensitivity is tightly tied to both sensory threshold and habituation. It refers to an individual’s responsiveness or reactivity to sensory stimuli from their internal or external environment, encompassing awareness or reactivity to lights, sounds, textures, smells, tastes, and movement. According to Dunn’s sensory processing model [6–8], habituation processes may modulate sensory threshold and, thus, affect sensory sensitivity. Specifically, an individual’s sensory profile results from the combination of two factors, structured along a continuum (“axes”): “neurological threshold” and “self-regulation”. Individuals with low thresholds exhibit heightened or faster reactivity to stimuli, even at minimal intensities (hypersensitivity). They may easily become overwhelmed and find intolerable even seemingly unnoticeable stimulations. Typically, they implement passive self-regulation strategies, experiencing discomfort and negative feelings. In contrast, those using active self-regulation strategies try to manage sensory inputs and engage in avoidance behaviors. In these cases, effective habituation processes can attenuate sensory sensitivity by decreasing responsiveness to repeated exposure, thereby preventing sensory overload, discomfort, and avoidance. Conversely, individuals with high sensory thresholds react to high-intensity stimuli (hyposensitivity). When employing passive self-regulation strategies, they may fail to notice or respond to sensory stimuli, appearing unmotivated [9, 10]. With active self-regulation strategies, they may seek sensory inputs or show interest in unusual sensory aspects of their environment. In these cases, slower habituation processes may facilitate stimulus detection. Importantly, different sensory profiles may co-occur in the same individual depending on the sensory modality, their status on that particular day (e.g., more rested or tired), or task requirements [7].

While Dunn defined “sensory sensitivity” as a specific pattern of sensory responsiveness including low sensory threshold and passive self-regulation strategies, in this manuscript, we refer to “sensory sensitivity” as a more general process of sensory responsiveness, which may also include high sensory threshold and active self-regulation strategies as well.

When the ability to regulate the response to sensory inputs in a graded manner, adapted to situational demands, is impaired, sensory sensitivity may be disrupted [11]. Recent research on scales assessing sensory processing suggests that sensory sensitivity is a continuous trait, with individuals falling into different sensitivity classes [12], which may vary uniquely for specific sensory modalities [13].

Altered sensory sensitivity patterns have been observed in various clinical populations, such as autism spectrum disorders (ASD, see [14] for a review), obsessive-compulsive disorder [15, 16], eating disorders [17, 18], attention-deficit/hyperactivity disorder [19], anxiety disorders [20], and chronic pain [21, 22]. In particular, abnormal sensory processing is prototypical of autistic people with ASD [23], who may exhibit both hyper- and hyposensitivity. For example, they may react adversely to noisy or visually complex environments, experience discomfort from tags or seams on clothing, and show aversion to unexpected touch [24–26]. On the other hand, they may appear indifferent to pain or uncomfortable temperatures while displaying excessive touching or smelling of objects and fascination by lights or movements [24–26]. Recent studies indicate that about 90% of ASD children had atypical sensory reactivity patterns in at least one sensory modality [27, 28]. Over-reactivity observed in ASD individuals was most evident when stimuli of different modalities (i.e., auditory and visual) occurred simultaneously, supporting the sensory gating hypothesis [29]. These patterns contribute to discomfort, anxiety, and stress, explaining a significant portion of their challenging behaviors and meltdowns [20, 27]. Consistent with the idea that sensory sensitivity is a continuous trait, altered sensitivity has been found to extend beyond individuals diagnosed with ASD to neurotypical individuals with autistic traits [30–35]. It is noteworthy that most disorders characterized by altered sensory sensitivity also exhibit atypical habituation processes [36], including ASD [37–42], obsessive-compulsive disorder [43], attention-deficit/hyperactivity disorder [44, 45], and chronic pain [46, 47]. For instance, individuals with ASD demonstrate slower attenuation of brain responses to repeated auditory, visual, or tactile stimuli compared to neurotypical controls, with abnormal habituation processing correlating with scores on autistic traits [37, 41] and sensory sensitivity [39–42]. These findings strongly support the link between sensory sensitivity and habituation processes [29, 43].

Despite this link appears straightforward, the individual habituation profile is not usually assessed as part of their sensory profile. This is partially due to the absence of practical tools for evaluating habituation in daily situations.

The main objective of this work was to validate an Italian version of the Sensory Habituation Questionnaire (S-Hab-Q). Originally developed in Hebrew and studied in an Israeli adult population [48], the questionnaire comprises questions aimed at gauging an individual’s typical adaptation time to sensory stimuli in daily life, particularly those that may potentially cause discomfort. Specifically, participants are asked to estimate the time they usually need for habituation or to ignore prolonged stimuli. We validated the questionnaire in a sample of healthy adults. In line with the original study, we assessed internal consistency and convergence validity by correlating questionnaire scores with those from a self-report instrument assessing sensory processing, the Sensory Perception Quotient (SPQ, [49]). Unlike the original study, we also tested the questionnaire’s factor structure based on sensory modality. Furthermore, given previous evidence that higher autistic traits reported by parents, rather than a clinical diagnosis, were associated with prolonged brain responses to sensory stimulation and decreased habituation [37], we examined correlations between the S-Hab-Q scores and the individual autistic traits using the Autism Quotient questionnaire (AQ, [50, 51]).

## Materials and methods

### Procedure

The survey was created in Google Forms and distributed through social networks and personal communications, using a snowball sampling method, from May to December 2021. Socio-demographic information, which included age, sex, nationality, education level, the presence of sensory deficits, medical diseases, and neurological or psychiatric disorders, was collected in an anonymous form. Data collection began after the person provided their written informed consent to participate. The whole research protocol conformed with the Declaration of Helsinki and was reviewed and approved by the Ethical Committee of the University of Palermo (n. 48/2021). The inclusion criteria comprised being older than 18 years old and a native Italian speaker.

### Participants

Participants were eligible if they did not report sensory impairments, current or previous psychiatric or neurological diagnoses, a diagnosis of neurodevelopmental disorders, or severe chronic medical conditions. More specifically, participants were asked if they have any sensory impairment (such as, visual, auditory, tactile, olfactory, gustative, or vestibular), and if they had ever had a diagnosis of psychiatric disorder (such as depression, anxiety, eating disorder, obsessive-compulsive disorder), neurological disorder (such as epilepsy, traumatic brain injury, or neurodegenerative diseases), neurodevelopmental disorder (such as autism, ADHD, learning disabilities, intellectual disability, language disorder, or motor disorder), or chronic medical conditions.

A total of 340 participants, aged 18 to 67 years, completed the survey. Seventy-eight participants were excluded for the following reasons: two were not-native Italian speakers, 24 reported sensory impairments (mainly visual and auditory), 4 had a neurological disorder diagnosis, 26 had anxiety or depressive disorders, 10 had obsessive-compulsive disorders, 10 had eating disorders, one had an autism spectrum disorder, and one had an intellectual disability. Consequently, the final sample- comprised 262 participants (mean age = 32.7 years, SD = 14.4, range = 18–67; females = 58.8%). Most participants (45%) held a secondary school diploma (equivalent to 8 years of education), while the remaining participants had completed primary school (5%), high school (30%), a bachelor’s degree (13%), or a master’s degree or higher (7%). All participants have Italian nationality.

### Questionnaires

#### Sensory Habituation Questionnaire (S-Hab-Q, [48])

The questionnaire consists of 25 items designed to assess the individual ability to adapt to external stimuli. Each question pertains to a specific sensory modality: visual (items 1, 4, 13, and 14), auditory (items 2, 8, 11, 15, 18, 19, and 22), tactile (items 3, 5, 6, 7, 10, 16, and 24), olfactory (9, 17, 20, and 23), gustative (12 and 21), or vestibular (item 25). The original S-Hab-Q exhibited high internal consistency (Cronbach’s alpha = .88) and demonstrated good convergent validity with other self-report measures on sensory processing, such as the Sensory Perception Quotient (SPQ [49]; r = .57, *p* < .001). We translated and adapted the English version of the questionnaire [48] into Italian. First, the items were translated by four native Italian speakers with expertise in psychology (V.T., P.T., L.M., N.P.). After reaching a consensus on each item, a native English speaker proficient in both languages back-translated the items into English. The final version of the questionnaire was reviewed and adjusted by the original authors (A.B.-S. and T.Y.P.). Some wording within the questions and the original 4-point rating scale were slightly modified. Namely, adverbs or references to temporal dimensions were removed from the questions and retained in the rating scale only. The final Likert scale was as follows: "pochissimo tempo" (very little time, 0), "poco tempo" (little time, 1), "molto tempo" (much time, 2), "moltissimo tempo" (very much time, 3). Consistent with the original questionnaire, the total score was computed as the sum of items, with a possible range of 0–75. Lower scores indicate faster/easier adaptation to stimuli, while higher scores indicate slower/more difficult adaptation. The full version of the questionnaire is available in the S1 Table.

#### Sensory Perception Quotient (SPQ, [49])

This tool is a 35-item questionnaire designed to assess self-reported hyper- and hyposensitivity to stimuli in adults. An example item is "I would be able to distinguish different people by their smell”. Each item is rated on a 4-point Likert scale, ranging from 0 (strongly agree) to 3 (strongly disagree). In the present study, the Italian translation by Brighenti & Keller, available at https://www.autismresearchcentre.com/tests, was used. To ensure consistency with the S-Hab-Q validation study [31] and improve the readability of SPQ scores, responses to items identifying hypersensitivity (n = 29) were reversed. The total score was computed as the sum of all items, so that higher scores indicate higher sensitivity and lower scores indicate lower sensitivity. Each item pertains to one of the following sensory domains: vision, hearing, touch, smell, and taste. The questionnaire demonstrated high internal consistency (Cronbach’s alpha = .93; [49]).

#### Autism-Spectrum Quotient (AQ, [50, 51])

This 50-item self-report questionnaire quantifies the degree of autistic traits of individuals [50, 51]. The AQ items assess the presence of core autistic symptoms across five subscales: social skills, attention switching, attention to detail, communication, and imagination. An example item is "I am often the last to understand the point of a joke”. Each item is rated on a four-point Likert scale, ranging from “definitely agree” to “definitely disagree”. A score of 1 is assigned for agreement with an ASD trait, while a score of 0 is assigned for disagreement. To minimize response bias, half of the items are reverse-scored. Higher scores indicate more autistic traits. The AQ has demonstrated robust predictive abilities in identifying individuals who receive a diagnosis of ASD in clinical settings. Additionally, it has exhibited specificity and sensitivity in non-clinical samples [52].

### Statistical analyses

The distribution of scores was examined, and normality tests were performed for the total scores of all questionnaires, as detailed in the S1 Fig and S2 Table. The S-Hab-Q and the AQ, but the SPQ, were positively skewed (Shapiro-Wilk’s *p*-values < .013), which indicates that some participants scored very high.

To test the internal consistency of the S-Hab-Q, Cronbach’s alpha (α) was computed. McDonald’s omega (ω) was also included, as it provides a more robust estimate of reliability when items are scored in a limited number of categories and do not contribute equally to the scale [53, 54].

A Confirmatory Factor Analysis (CFA) was conducted to test a factor model based on a sensory modality categorization of items. First, a four-latent variables structure was considered, with a factor for each sensory modality, namely, vision, hearing, touch, smell and taste. The scores of the items assessing smell and taste were averaged to optimize the number of items in the subscale and based on the strong correlation between these two senses (ρ = .403, *p* < .001). The item assessing vestibular sensitivity (n = 25) was removed to avoid basing a factor on a one-item scale. A robust diagonally weighted least squares (DWLS) estimator for ordinal items (e.g., Likert-type scales) was used for the CFA. To evaluate the overall model fit, the following goodness of fit indices were computed: the comparative fit index (CFI), the Tucker-Lewis index (TLI), the root mean square error of approximation (RMSEA), and the Standardized Root Mean Square Residual (SRMR). To reach a good fit, the CFI and TLI indexes should exceed .95, whereas the RMSEA should be lower than .06, and the SMSR should be lower than .08 [55]. A set of 1000 bootstrap samples was used for computing the standard errors of the parameter estimates.

Convergent validity was assessed by conducting correlational analyses between the total S-Hab-Q scores (including item 25), the mean scores of each S-Hab-Q factor, and the SPQ scores. Given some questionnaires’ scores slightly deviated from normal distribution non-parametric correlations were performed (Spearman’s rho coefficient, ρ). The False Discovery Rate (FDR) method to control for multiple comparisons was applied when appropriate [56].

All the analyses were performed in R (R Core Team, 2015), using the *psych* [57] and *lavaan* [58] packages. No missing values were present as all questions were compulsory in the form. Unless otherwise specified, raw, non-transformed questionnaire scores were analyzed.

## Results

Descriptive statistics of the questionnaires’ scores are reported in S2 Table. The mean S-Hab-Q total score was 24.47 (SD = 8.77, range = 5–63). The mean S-Hab-Q item scores ranged from .366 (SD = .596) for “I continue to feel dizzy after I got off the elevator or escalator” (3% of participants answered 2, much time, or 3, very much time) to 1.847 (SD = .767) for “When there is a certain smell (for example garbage, cigarettes, sweat, laundry or perfume) I continue to smell it even after being in the room” (70.2% of participants answered 2, much time, or 3, very much time).

### Internal consistency

Overall, the S-Hab-Q showed a high internal consistency, as revealed by Cronbach’s alpha (α = .83, 95% CI [.79, .87]) and McDonald’s omega (ω = .85). The correlation coefficients between the items grouped according to sensory modality ranged from low, between vision and smell & taste (r = .251, *p* < .001), to moderately high, between touch and smell & taste (r = .61, *p* < .001; see S3 Table for the full correlation matrix).

### Factor analysis

**Fig 1:**
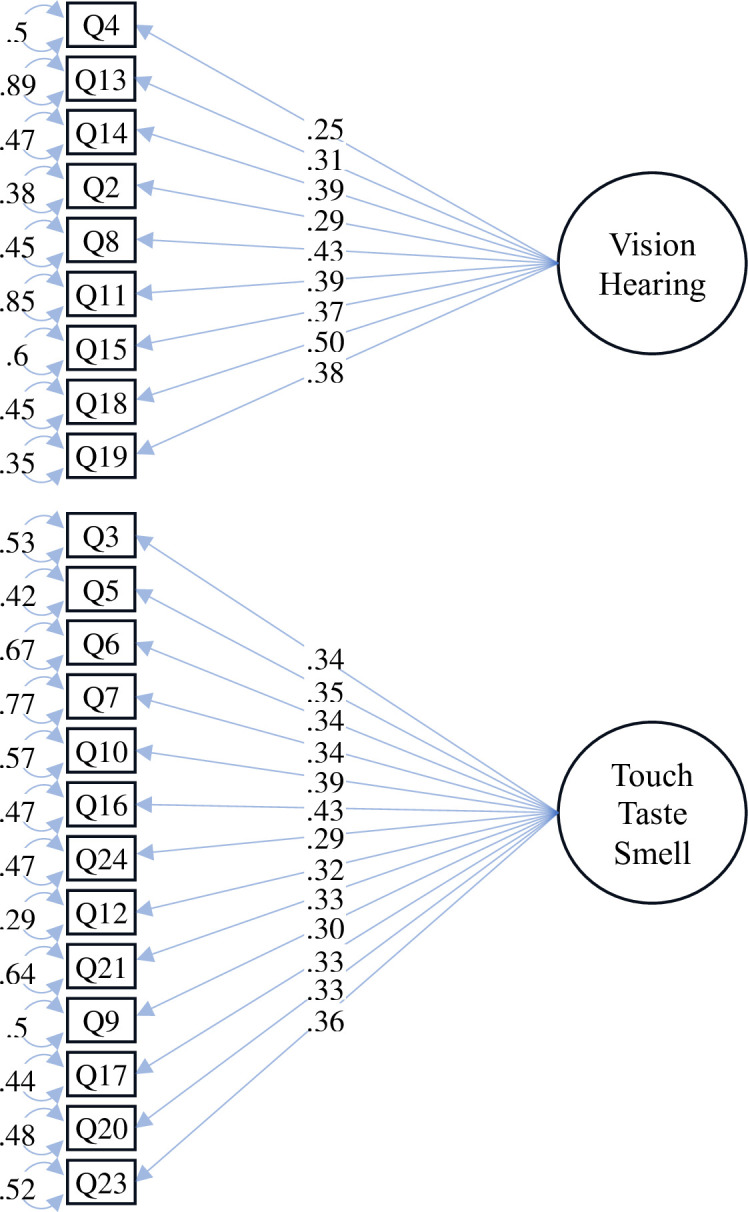
Path diagram of the 2-factor CFA analysis.

First, a CFA with four latent variables, corresponding to the visual, auditory, tactile, and smell and taste modalities, was performed. This four-factor model, represented in S2 Fig, exhibited satisfactory goodness-of-fit indexes (χ^2^(246) = 325.37, *p* = .001, CFI = .957, TLI = .951, RMSEA = .035, 90% CI [.024, .045], SRMR = .071]. Given the high covariance between the vision and hearing factors (r = 1.02, SE = .123, *p* < .001), and between touch, smell, and taste (r = .997, SE = .058, *p* < .001), a two-factor model was tested as well, which included a visual-auditory factor and a tactile-taste-smell factor (Fig 1). The latter model better fitted the data (χ^2^(251) = 327.24, *p* = .001, CFI = .958, TLI = .954, RMSEA = .034, 90% CI [.023, .044], SRMR = .071).The internal consistency of each factor was good as well (α_vision,hearing_ = .72, 95% CI [.65, .77], α_touch,smell,taste_ = .75, 95% CI [.69, .79]; ω_vision,hearing_ = .79, ω_touch,smell,taste_ = .78).

### Convergent validity

A positive Spearman’s coefficient between the S-Hab-Q and the SPQ total scores emerged (ρ(262) = .32, *p* < .001). When considering the sensory modality, the mean scores of the SPQ positively correlated with the mean scores of the S-Hab-Q factors, although these were mild to moderate (see Table 1).

**Table 1:** Spearman’s correlation coefficients between mean scores of each S-Hab-Q factor and mean scores of the corresponding sensory modalities of the SPQ.

		S-Hab-Q	
		Vision, Hearing	Touch, Smell, Taste
**SPQ**	**Vision**	.338***	.305***
	**Hearing**	.180***	.208**
	**Touch**	.239***	.360***
	**Smell**	.038	.269***
	**Taste**	.014	.078

S-Hab-Q, Sensory Habituation Questionnaire; SPQ, Sensory Perception Quotient

** *p* < .01

*** *p* < .001. All significant comparisons survived the FDR correction.

### Habituation and autistic traits

A positive correlation between the S-Hab-Q total score and the AQ total score was found (ρ(262) = .328, *p* < .001). More specifically, the S-Hab-Q total score positively correlated with three AQ subscales, namely, social skills, attention switching, and communication (see Table 2 for a full report). These results were further confirmed by a median split analysis. Participants were divided into two groups based on their S-Hab-Q scores: low and high, according to the median value of 24. The Mann-Whitney test revealed that participants with high S-Hab-Q scores had significantly higher AQ scores (Z = 5.1, *p* < .001).

**Table 2:** Spearman’s correlation coefficients between mean scores of each S-Hab-Q factor and the AQ subscales.

		S-Hab-Q	
		Vision, Hearing	Touch, Smell, Taste
**AQ**	**Total**	.292***	.289***
	**Social skill**	.250***	.178**
	**Attention switching**	.306***	.220***
	**Attention to details**	-.019	.161**
	**Communication**	.248***	.212***
	**Imagination**	.099	.054

S-Hab-Q, Sensory Habituation Questionnaire; AQ, Autism Quotient

** *p* < .01

*** *p* < .001. All significant comparisons survived the FDR correction.

**Fig 2:**
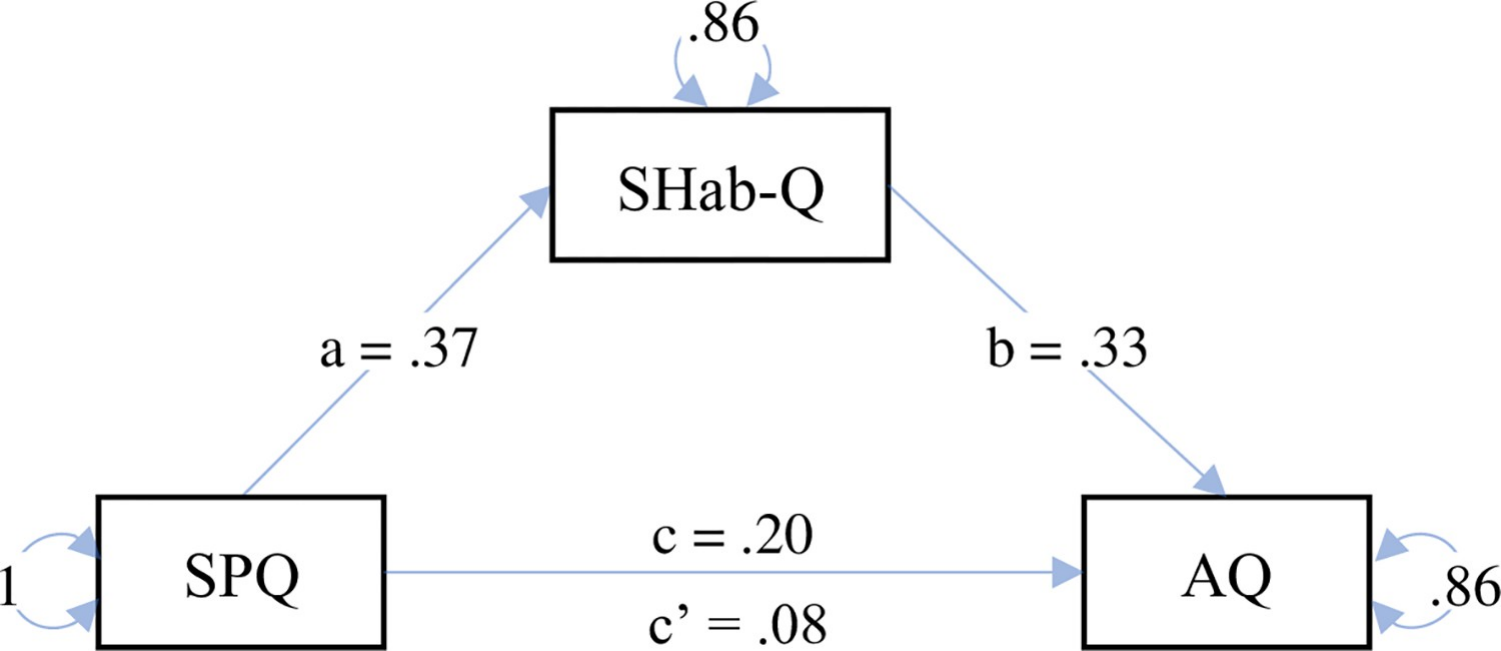
Mediation analysis. The analysis considered two models: AQ ~ S-Hab-Q + SPQ and S-Hab-Q ~ SPQ. The coefficient *c* represents the total effect of SPQ on AQ; *c’* represents the direct effect of SPQ on AQ partialling out the effect of S-Hab-Q (c’ = c–ab). The mediation effect is *ab* and represents the indirect effect of SPQ on AQ through S-Hab-Q.

To verify the presence of hypersensitivity in people with autistic traits, a correlation was performed between the SPQ and AQ scores. As expected, this correlation was positive and significant, although mild (ρ(262) = .153, *p* = .013). To explore the extent to which autistic traits were explained by sensory sensitivity and/or by sensory habituation, a regression analysis (general linear model) was conducted with the AQ score as the dependent variable and the SPQ and S-Hab-Q scores as predictors (AQ ~ S-Hab-Q + SPQ). The result revealed that only the S-Hab-Q score predicted the AQ, not the SPQ score (see S4 Table). This finding was further investigated through a mediation analysis, with the hypothesis that S-Hab-Q mediates the association between SPQ and AQ (i.e., AQ ~ SPQ + (S-Hab-Q)). The results, represented in Fig 2 and fully reported in S5 Table, confirmed this hypothesis. While there was no direct effect of SPQ on AQ, there was a significant indirect effect of SPQ on AQ, mediated by the Shab-Q. To explore the contribution of S-Hab-Q in mediating the relationship between SPQ and AQ, we performed the mediation analysis separately for each AQ subscale. The results are reported in S3–S7 Figs and S6–S10 Tables. The S-Hab-Q mediated the relationship in the social skill, attention switching, and communication subscales of AQ, but not in the attention to detail and imagination subscales.

### Demographic correlates

An independent-sample t-test showed significant differences in S-Hab-Q scores by sex. On average, females reported higher scores than males (M_female_ = 25.77, SD = 9.15; M_male_ = 22.60, SD = 7.86; t(260) = 3.17, *p* = .004). Furthermore, the Spearman’s correlation between the S-Hab-Q total score and age was negative (ρ(262) = –.24, *p* = .042). Median S-Hab-Q scores did not differ across education levels (χ^2^ = 3.20, *p* = .363). To better examine the effect of sex and age, and their interaction, we provide demographic variables and descriptive statistics of the questionnaires’ total scores grouped by sex (S11 and S12 Tables). Furthermore, both factors were included as predictors of the S-Hab-Q scores in a linear regression model (S-Hab-Q ~ Sex × Age). The analysis revealed that sex, but not age, significantly affected the S-Hab-Q scores (see S13 Table). Subsequently, to understand the moderating effect of sex on the relationship between the S-Hab-Q and the SPQ scores, as well as between the S-Hab-Q and the AQ scores, we entered sex as a factor in two separate models (S-Hab-Q ~ Sex × SPQ and S-Hab-Q ~ Sex × AQ). This analysis revealed that sex significantly interacted with both SPQ and AQ scores (see S13 Table). Namely, the positive correlation between S-Hab-Q and SPQ, as well as S-Hab-Q and AQ, exhibited a steeper slope for females compared to males (see S8 Fig). Lastly, we explored sex differences in the mediation model by analyzing the data separately for each sex (S9 and S10 Figs and S14 and S15 Tables). The results showed that in males the mediation S-Hab-Q did not significantly contributed to explaining the weak relationship between SPQ and AQ.

## Discussion

In this study, we adapted and validated an Italian version of the Sensory Habituation Questionnaire (S-Hab-Q), originally developed by Podoly and Ben-Sasson (2020) in an Israeli sample of healthy adults [48]. The S-Hab-Q is a self-report tool assessing the ability to adapt to sensory stimulation in daily life, [48] with a specific focus on mapping individual difficulty in suppressing responses to repeated or prolonged daily sensory stimulation. Our primary aim was to replicate the findings of the original validation study, including verifying internal consistency and examining the relationship between the S-Hab-Q scores and scores on a questionnaire assessing sensory sensitivity, the Sensory Perception Quotient questionnaire (SPQ). Our secondary aims were to evaluate the goodness-of-fit of a modality-based factor model and explore the relationship between habituation profiles and autistic traits.

Overall, the S-Hab-Q demonstrated good internal consistency (α = .83), comparable to the original version (α = .88). The mean total score (24.47) closely matched the original findings (23.66), reinforcing the questionnaire’s reliability across different cultural contexts. The significant correlations between the S-Hab-Q and the SPQ scores replicated the original validation study and provided further support to the notion that slower habituation (higher S-Hab-Q scores) is associated with hypersensitivity. This result is in line with previous research suggesting that impaired habituation processes may alter the modulation of sensory processing [6–8] Individuals with slow habituation processes experience prolonged sensory stimulation, which lead them to feel overwhelmed and avoid further exposure. In contrast, faster habituation processes are associated with hyposensitivity and sensory-seeking behaviors. Of note, the correlation values with the SPQ were not particularly high (*ρ* = .318), suggesting that the two questionnaires assess some common mechanisms but also capture unique aspects associated with sensory processing.

The CFA analysis revealed that a two-factor model based on the sensory modalities explains the S-Hab-Q scores with a satisfactory goodness-of-fit. The two factors corresponded to 1) vision and hearing, and 2) touch, smell, and taste. This classification might have reflected a dispositional multisensory integration process across senses. Visual and auditory information are often bound together (e.g., in speech perception), as well as taste emerges from the synthesis of gustatory, olfactory, and tactile information [59–62]. Furthermore, compared to visual and auditory systems, which are more externally oriented, olfactory, gustatory, and tactile systems are more closely tied to bodily sensations.

The significant correlation between the S-Hab-Q and the AQ scores revealed that a higher presence of autistic traits was associated with slower sensory habituation in neurotypical adults. Specifically, the AQ subscales on social skills, attention/switching, and communication AQ scales exhibited positive and significant correlations with the S-Hab-Q score. This result aligns with previous laboratory research showing impaired habituation processes in ASD individuals, particularly with auditory stimuli (e.g., [37–39, 42]). Interestingly, studies have shown that reduced repetition suppression of responses to auditory [63, 64] and tactile [65] stimuli in early life can predict future ASD diagnosis in infants at risk for autism. The AQ scales that positively correlated with the S-Hab-Q score in our study also showed significant correlations with the sensory sensitivity scored with the Adult/Adolescent Sensory Profile questionnaire, both in high-functioning ASD and typically developing adults, as reported by Mayer [28]. Moreover, our result are in line with Jamal et al.’s [41] study, which demonstrated significant correlations between the lack of modulatory effects in the neurophysiological habituation mechanisms in autistic children and the parents’ reports on social communication difficulties. Overall, these findings remark the link between autistic behaviors and altered sensory habituation.

The mediation analysis clarified that the link between sensory sensitivity and autistic traits is mediated by habituation responses. This result suggests that sensoriality and autistic traits may not be directly related but associated through a third variable, the individual habituation profile. In the light of the Bayesian predictive framework, the autistic brain may not effectively attenuate bottom-up signals by top-down expectations [66, 67]. This impaired top-down modulation might impact habituation processes and, ultimately, sensory sensitivity. Although alternative hypotheses could account for the altered habituation responses, in both atypical [68] and neurotypical [34] populations, our study provides robust evidence that recognizing individual differences in habituation can elucidate the source of heterogeneity in sensory profiles among individuals with autistic traits, and ultimately improve personalized interventions. Tailoring interventions to accommodate specific habituation and sensory sensitivities can help create less distressing learning environments, as well as more effective healthcare experiences, workplaces, and recreational facilities. Ultimately, this approach can help reduce challenging behaviors and stress, leading to an improvement in their overall quality of life.

Some differences in the data collected with the original S-Hab-Q version should be noted. First, in our study, items related to vestibular and smell modalities received the lowest and highest scores, respectively. This contrasts with the original Israeli sample, where touch and hearing modalities showed the lowest and highest scores. These differences suggest potential cultural or sample-related variations in sensory habituation profiles across different populations.

Second, a sex difference was found in the Italian but not in the Israeli study. Specifically, in our study, female participants showed higher scores, indicating slower habituation to stimuli compared to male participants. This finding is consistent with evidence of a higher self-reported sensory sensitivity among females in various sensory domains [69]. Moreover, our results suggest that the relationship between sensory habituation and sensory sensitivity, and autist traits may be more pronounced in females than males, and that habituation processes appear to poorly mediate the relationship between sensory sensitivity and autistic traits among males. These findings underscore the need for further investigations to understand the sensory processing differences across sexes and their implications for individuals with autistic traits.

Our study revealed also that older age was more associated with faster habituation. This correlation aligns with the original validation of the S-Hab-Q and supports existing evidence suggesting that sensory threshold increase with age [70]. However, our analysis showed that age does not explain a significant proportion of variance in S-Hab-Q scores when sex moderates their association.

The lack of education level differences in our study may be ascribed, at least in part, to a lower educational level within our Italian sample compared to the Israeli sample. Another source of variability could be the modified Likert scale of the Italian version, which emphasized the temporal dimension in judging the duration of sensory adaptation.

Some methodological and conceptual limitations of the work should be acknowledged. First, the use of different questionnaires such as the SPQ and AQ might have led to different correlations between habituation, sensory sensitivity, and autistic traits. These differences could potentially influence the interpretation of how sensory habituation relates to sensory sensitivity and autistic traits. Additionally, while our study employed a modality-based factor analysis to classify sensory habituation, an exploratory analysis might have detected other factors not related to the sensory modality classification. The study would benefit from the cross-validation of the new Likert scale labels on an independent Italian sample, as well as an assessment of test-retest reliability, to ensure the generalizability of our findings and their consistency over time. Lastly, although the S-Hab-Q provides an estimate of individual habituation levels in daily life, further research is warranted to validate whether this self-reported measure correlates with more objective habituation and sensitivity testing.

## Conclusions

The Italian version of the S-Hab-Q is a valid tool for measuring sensory habituation to stimuli in everyday environments. Its use may have important implications for understanding how sensory stimuli impact an individual’s daily life. This information could be valuable for properly assessing sensory processing disorders and tailoring personalized interventions that help people improve their quality of life, in particular in individuals in the general population with higher levels of autistic traits.

## Supporting information

S1 Table The Italian Sensory Habituation Questionnaire (S-Hab-Q). The validated questionnaire.
(DOCX)

S2 Table Descriptive statistics and normality tests of the questionnaires’ total scores. (DOCX)

S3 Table Correlation analysis. Spearman’s coefficients of the correlations between mean scores of the S-Hab-Q items grouped by sensory modality.
(DOCX)

S4 Table Regression analysis. (DOCX)

S5 Table Mediation analysis. The output obtained with the *lavaan* package is reported. The 95% Confidence Interval (CI) was computed on 5000 iterations. The combination of SPQ and S-Hab-Q explains the 13.5% of the AQ variance (R2).
(DOCX)

S6 Table Mediation model table for the AQ social skill subscale. The R^2^ values refer to the combination of SPQ and S-Hab-Q in explaining the dependent variable.
(DOCX)

S7 Table Mediation model for the attention switching AQ subscale. (DOCX)

S8 Table Mediation model for the attention to detail AQ subscale. (DOCX)

S9 Table Mediation model for the communication AQ subscale. (DOCX)

S10 Table Mediation model for the imagination AQ subscale. (DOCX)

S11 Table Demographic variables grouped by sex. (DOCX)

S12 Table Descriptive statistics of the questionnaires’ total scores grouped by sex. (DOCX)

S13 Table Regression models. In the first model, sex and age factors were entered as predictors of the S-Hab-Q score. The factor sex was entered as predictor in the models predicting the S-Hab-Q score from the SPQ score and the S-Hab-Q score from the AQ score.
(DOCX)

S14 Table Mediation model table in females. The R^2^ values refer to the combination of SPQ and S-Hab-Q in explaining the dependent variable.
(DOCX)

S15 Table Mediation model table in males. (DOCX)

S1 Fig Distribution of the questionnaires’ scores. Probability density function (left panel) and quantile-quantile plot (q-q plot, right panel) of the questionnaires’ total scores.
(DOCX)

S2 Fig Path diagram of the four-factor CFA analysis. (DOCX)

S3 Fig Mediation model diagram for the AQ social skill subscale. The analysis considered two models: AQ ~ S-Hab-Q + SPQ and S-Hab-Q ~ SPQ. The coefficient *c* represents the total effect of SPQ on AQ; *c’* represents the direct effect of SPQ on AQ partialling out the effect of S-Hab-Q (c’ = c–ab). The mediation effect is *ab* and represents the indirect effect of SPQ on AQ through S-Hab-Q.
(DOCX)

S4 Fig Mediation model for the attention switching AQ subscale. (DOCX)

S5 Fig Mediation model for the attention to detail AQ subscale. (DOCX)

S6 Fig Mediation model for the communication AQ subscale. (DOCX)

S7 Fig Mediation model for the imagination AQ subscale. (DOCX)

S8 Fig Relationship between questionnaires’ scores grouped by sex. (DOCX)

S9 Fig Mediation model diagram in females. The analysis considered two models: AQ ~ S-Hab-Q + SPQ and S-Hab-Q ~ SPQ. The coefficient *c* represents the total effect of SPQ on AQ; *c’* represents the direct effect of SPQ on AQ partialling out the effect of S-Hab-Q (c’ = c–ab). The mediation effect is *ab* and represents the indirect effect of SPQ on AQ through S-Hab-Q.
(DOCX)

S10 Fig Mediation model diagram in males. (DOCX)
