# Supplementary material for: Measuring habituation to stimuli: The Italian version of the Sensory Habituation Questionnaire
Source: PLoS One. 2024 Dec 31;19(12):e0309030. doi: 10.1371/journal.pone.0309030 (PMC11687914; doi:10.1371/journal.pone.0309030)
Supplement: S1 Table — The validated questionnaire. (DOCX) [file pone.0309030.s001.docx]

**S1 Table. The Italian Sensory Habituation Questionnaire (S-Hab-Q).** The validated questionnaire.

*Gli individui differiscono tra di loro nella capacità di adattarsi agli stimoli sensoriali e nel continuare a svolgere le proprie attività in presenza di un particolare stimolo. In questo questionario le viene chiesto di pensare quanto tempo le serve per abituarsi a stimoli diversi.*

|  | **pochissimo tempo** | **poco tempo** | **molto tempo** | **moltissimo tempo** |
| --- | --- | --- | --- | --- |
| 1. La mattina per abituarmi alla luce del sole ho bisogno di … |  |  |  |  |
| 1. Per concentrarmi sul quello che sto facendo mentre nella stanza è presente un ventilatore rumoroso ho bisogno di … |  |  |  |  |
| 1. Continuo a sentire il contatto con orologio/anello/cappello/occhiali da sole/collana sulla mia pelle ogni volta che li indosso per… |  |  |  |  |
| 1. Quando la luce di una stanza è troppo forte non riesco a leggere per … |  |  |  |  |
| 1. Dopo che qualcuno mi tocca riesco a sentire il punto dove sono stato toccato sul mio corpo per … |  |  |  |  |
| 1. Dopo essere andato dal parrucchiere continuo a sentire pezzi di capelli sul mio collo, anche quando non ce ne sono più, per … |  |  |  |  |
| 1. Non riesco a sostenere la conversazione se qualcuno mi tocca mentre parla per … |  |  |  |  |
| 1. Continuo a sentire il rumore dell’aria condizionata, anche dopo che è accesa da un po’, per … |  |  |  |  |
| 1. Quando sento l’odore di cibo non riesco a concentrarmi, anche se non sono affatto affamato, per … |  |  |  |  |
| 1. Se stringo i lacci delle scarpe più forti in un piede continuo a sentire la differenza tra i piedi per … |  |  |  |  |
| 1. Per addormentarmi in presenza di un orologio che ticchetta o di acqua che gocciola mi ci vuole ... |  |  |  |  |
| 1. Dopo aver mangiato qualcosa di acido/salato/dolce, il sapore acido/salato/dolce mi rimane in bocca per … |  |  |  |  |
| 1. Se la stanza non è completamente al buio per addormentarmi ci impiego … |  |  |  |  |
| 1. Quando lavoro al computer sono consapevole dello sfarfallio dello schermo per … |  |  |  |  |
| 1. Per concentrarmi in un ambiente rumoroso ho bisogno di … |  |  |  |  |
| 1. Dopo aver toccato una sostanza (ad esempio colla, vernice, sabbia) continuo a sentire questa sensazione sul mio corpo per … |  |  |  |  |
| 1. Quando una persona indossa un profumo trovo difficile concentrarmi sulla conversazione con quella persona per … |  |  |  |  |
| 1. Mentre lavoro al computer continuo a sentire il rumore del processore per … |  |  |  |  |
| 1. Mentre accendo la TV sento i beep/suoni che emette per … |  |  |  |  |
| 1. Se nella stanza in cui entro è presente un certo odore (ad esempio spazzatura, sigarette, Sudore, lavanderia o profumo) continuo a sentirlo per … |  |  |  |  |
| 1. Quando mangio cibo con una consistenza irregolare (ad esempio yogurt con muesli o zuppa con pezzi di verdure) sono sorpreso del fatto che la sua consistenza nella mia bocca cambi ad ogni morso per ... |  |  |  |  |
| 1. In presenza di suoni (TV, musica) che provengono dalla stanza a fianco, non riesco a mangiare per … |  |  |  |  |
| 1. Continuo a sentire gli odori in cucina. dopo che la cottura è finita, per … |  |  |  |  |
| 1. Continuo a sentire il tocco dell’erba sul mio corpo, anche se non sono più su un prato, per … |  |  |  |  |
| 1. Continuo a sentire capogiri dopo essere sceso da un ascensore o da scale mobili per … |  |  |  |  |
