# Supplementary material for: Measuring habituation to stimuli: The Italian version of the Sensory Habituation Questionnaire
Source: PLoS One. 2024 Dec 31;19(12):e0309030. doi: 10.1371/journal.pone.0309030 (PMC11687914; doi:10.1371/journal.pone.0309030)
Supplement: S2 Table — (DOCX) [file pone.0309030.s002.docx]

| **Variables** | **Mean** | **SD** | **Median** | **Min** | **Max** | **Skew** | **Kurtosis** | **Shapiro-Wilk** | |
| --- | --- | --- | --- | --- | --- | --- | --- | --- | --- |
|  |  |  |  |  |  |  |  | *W* | *p* |
| **S-Hab-Q** | 24.47 | 8.77 | 24 | 5 | 63 | 1.1 | .54 | .98 | < .001 |
| **SPQ** | 57.66 | 11.02 | 57 | 24 | 94 | .21 | .27 | .99 | .211 |
| **AQ** | 18.13 | 5.65 | 18 | 3 | 39 | .4 | .37 | .98 | .013 |
| **Age** | 32.69 | 14.41 | 25 | 18 | 67 | .15 | -.64 | .81 | < .001 |

**S2 Table.** **Descriptive statistics and normality tests of the questionnaires’ total scores.**

S-Hab-Q, Sensory Habituation Questionnaire; SPQ, Sensory Perception Quotient; AQ, Autism Quotient.
