# Supplementary material for: Measuring habituation to stimuli: The Italian version of the Sensory Habituation Questionnaire
Source: PLoS One. 2024 Dec 31;19(12):e0309030. doi: 10.1371/journal.pone.0309030 (PMC11687914; doi:10.1371/journal.pone.0309030)
Supplement: S3 Table — Spearman’s coefficients of the correlations between mean scores of the S-Hab-Q items grouped by sensory modality. (DOCX) [file pone.0309030.s003.docx]

**S3 Table.** **Correlation analysis.** Spearman’s coefficients of the correlations between mean scores of the S-Hab-Q items grouped by sensory modality.

|  | | **S-Hab-Q** | | | |
| --- | --- | --- | --- | --- | --- |
|  | | **Vision** | **Hearing** | **Touch** | **Smell & Taste** |
| **S-Hab-Q** | **Vision** | 1 | .487*** | .314*** | .251*** |
|  | **Hearing** |  | 1 | .48*** | .450*** |
|  | **Touch** |  |  | 1 | .610*** |
|  | **Smell & Taste** |  |  |  | 1 |

S-Hab-Q, Sensory Habituation Questionnaire; ** p < .01, *** p < .001. All *p*-values survived the FDR correction for multiple comparisons.
