# Supplementary material for: Measuring habituation to stimuli: The Italian version of the Sensory Habituation Questionnaire
Source: PLoS One. 2024 Dec 31;19(12):e0309030. doi: 10.1371/journal.pone.0309030 (PMC11687914; doi:10.1371/journal.pone.0309030)
Supplement: S4 Table — (DOCX) [file pone.0309030.s004.docx]

**S4 Table. Regression analysis.**

| **Model** | **B (SE)** | **t** | ***p*** |
| --- | --- | --- | --- |
| AQ ~ S-Hab-Q + SPQ | | | |
| S-Hab-Q | .21 (.04) | 5.31 | **< .001** |
| SPQ | .04 (.03) | 1.27 | .204 |
| R^2^ = .13 |  |  |  |

S-Hab-Q, Sensory Habituation Questionnaire; SPQ, Sensory Perception Quotient; AQ, Autism Quotient.
