# Supplementary material for: Measuring habituation to stimuli: The Italian version of the Sensory Habituation Questionnaire
Source: PLoS One. 2024 Dec 31;19(12):e0309030. doi: 10.1371/journal.pone.0309030 (PMC11687914; doi:10.1371/journal.pone.0309030)
Supplement: S5 Table — The output obtained with the lavaan package is reported. The 95% Confidence Interval (CI) was computed on 5000 iterations. The combination of SPQ and S-Hab-Q explains the 13.5% of the AQ variance (R2). (DOCX) [file pone.0309030.s005.docx]

**S5 Table.** **Mediation analysis**. The output obtained with the *lavaan* package is reported. The 95% Confidence Interval (CI) was computed on 5000 iterations. The combination of SPQ and S-Hab-Q explains the 13.5 % of the AQ variance (R^2^).

|  | **Coefficient** | **β (SE)** | **z** | ***p*** | **Lower CI** | **Upper CI** |
| --- | --- | --- | --- | --- | --- | --- |
| AQ social skill ~ S-Hab-Q | b | .33 (.07) | 4.78 | **< .001** | .19 | .46 |
| AQ social skill ~ SPQ | c | .08 (.06) | 1.29 | .198 | -.05 | .19 |
| S-Hab-Q ~ SPQ | a | .37 (.06) | 6.52 | **< .001** | .26 | .48 |
| Indirect effect | ab | .12 (.03) | 3.41 | **.001** | .06 | .20 |
| Total effect | ab + c | .20 (.07) | 2.94 | **.002** | .06 | .33 |
| R^2^ = .13 |  |  |  |  |  |  |

AQ, Autism Quotient; S-Hab-Q, Sensory Habituation Questionnaire; SPQ, Sensory Perception Quotient.
