# Supplementary material for: Measuring habituation to stimuli: The Italian version of the Sensory Habituation Questionnaire
Source: PLoS One. 2024 Dec 31;19(12):e0309030. doi: 10.1371/journal.pone.0309030 (PMC11687914; doi:10.1371/journal.pone.0309030)
Supplement: S6 Table — The R2 values refer to the combination of SPQ and S-Hab-Q in explaining the dependent variable. (DOCX) [file pone.0309030.s006.docx]

**S6 Table. Mediation model table for the AQ social skill subscale.**

|  | **Coefficient** | **β (SE)** | **z** | ***p*** | **Lower CI** | **Upper CI** |
| --- | --- | --- | --- | --- | --- | --- |
| AQ social skill ~ S-Hab-Q | b | .31 (.06) | 4.50 | **< .001** | .17 | .44 |
| AQ social skill ~ SPQ | c | -.14 (.06) | -2.09 | **.036** | -.28 | -.01 |
| S-Hab-Q ~ SPQ | a | .37 (.05) | 6.51 | **< .001** | .25 | .48 |
| Indirect effect | ab | .11 (.03) | 3.25 | **.001** | .05 | .19 |
| Total effect | ab + c | -.03 (.08) | -.36 | .716 | -.18 | .12 |
| R^2^ = .08 |  |  |  |  |  |  |
