# Supplementary material for: Measuring habituation to stimuli: The Italian version of the Sensory Habituation Questionnaire
Source: PLoS One. 2024 Dec 31;19(12):e0309030. doi: 10.1371/journal.pone.0309030 (PMC11687914; doi:10.1371/journal.pone.0309030)
Supplement: S7 Table — (DOCX) [file pone.0309030.s007.docx]

**S7 Table. Mediation model for the attention switching AQ subscale.**

|  | **Coefficient** | **β (SE)** | **z** | ***p*** | **Lower CI** | **Upper CI** |
| --- | --- | --- | --- | --- | --- | --- |
| AQ attention switching ~ S-Hab-Q | b | .35 (.06) | 6.00 | **< .001** | .23 | .46 |
| AQ attention switching ~ SPQ | c | -.01 (.06) | -.23 | .816 | -.14 | .11 |
| S-Hab-Q ~ SPQ | a | .37 (.05) | 6.66 | **< .001** | .26 | .48 |
| Indirect effect | ab | .13 (.03) | 3.92 | **<.001** | .06 | .20 |
| Total effect | ab + c | .12 (.06) | 1.69 | .091 | -.02 | .25 |
| R^2^ = .12 |  |  |  |  |  |  |
