# Supplementary material for: Measuring habituation to stimuli: The Italian version of the Sensory Habituation Questionnaire
Source: PLoS One. 2024 Dec 31;19(12):e0309030. doi: 10.1371/journal.pone.0309030 (PMC11687914; doi:10.1371/journal.pone.0309030)
Supplement: S8 Table — (DOCX) [file pone.0309030.s008.docx]

**S8 Table. Mediation model for the attention to detail AQ subscale.**

|  | **Coefficient** | **β (SE)** | **z** | ***p*** | **Lower CI** | **Upper CI** |
| --- | --- | --- | --- | --- | --- | --- |
| AQ attention to detail ~ S-Hab-Q | b | -.08 (.06) | -1.12 | .262 | -.20 | .06 |
| AQ attention to detail ~ SPQ | c | .39 (.05) | 7.46 | **< .001** | .28 | .49 |
| S-Hab-Q ~ SPQ | a | .37 (.05) | 6.52 | **< .001** | .25 | .48 |
| Indirect effect | ab | -.03 (.02) | -1.10 | .271 | -.08 | .02 |
| Total effect | ab + c | .36 (.05) | 7.67 | **< .001** | .26 | .45 |
| R^2^ = .13 |  |  |  |  |  |  |
