# Supplementary material for: Measuring habituation to stimuli: The Italian version of the Sensory Habituation Questionnaire
Source: PLoS One. 2024 Dec 31;19(12):e0309030. doi: 10.1371/journal.pone.0309030 (PMC11687914; doi:10.1371/journal.pone.0309030)
Supplement: S9 Table — (DOCX) [file pone.0309030.s009.docx]

**S9 Table. Mediation model for the communication AQ subscale.**

|  | **Coefficient** | **β (SE)** | **z** | ***p*** | **Lower CI** | **Upper CI** |
| --- | --- | --- | --- | --- | --- | --- |
| AQ communication ~ S-Hab-Q | b | .31 (.07) | 4.51 | **< .001** | .16 | .45 |
| AQ communication ~ SPQ | c | -.02 (.06) | -.24 | .810 | -.14 | .11 |
| S-Hab-Q ~ SPQ | a | .37 (.05) | 6.56 | **< .001** | .26 | .48 |
| Indirect effect | ab | .11 (.03) | 3.19 | **.001** | .05 | .19 |
| Total effect | ab + c | .10 (.07) | 1.33 | .184 | -.05 | .24 |
| R^2^ = .09 |  |  |  |  |  |  |
