# Supplementary material for: Measuring habituation to stimuli: The Italian version of the Sensory Habituation Questionnaire
Source: PLoS One. 2024 Dec 31;19(12):e0309030. doi: 10.1371/journal.pone.0309030 (PMC11687914; doi:10.1371/journal.pone.0309030)
Supplement: S10 Table — (DOCX) [file pone.0309030.s010.docx]

**S10 Table. Mediation model for the imagination AQ subscale.**

|  | **Coefficient** | **β (SE)** | **z** | ***p*** | **Lower CI** | **Upper CI** |
| --- | --- | --- | --- | --- | --- | --- |
| AQ imagination ~ S-Hab-Q | b | .09 (.06) | 1.44 | .150 | -.03 | .22 |
| AQ imagination ~ SPQ | c | -.06 (.07) | -.82 | .410 | -.19 | .07 |
| S-Hab-Q ~ SPQ | a | .37 (.05) | 6.59 | **< .001** | .25 | .48 |
| Indirect effect | ab | .03 (.02) | 1.35 | .176 | -.01 | .09 |
| Total effect | ab + c | -.02 (.06) | -.34 | .733 | -.15 | .09 |
| R^2^ = .01 |  |  |  |  |  |  |
