# Supplementary material for: Measuring habituation to stimuli: The Italian version of the Sensory Habituation Questionnaire
Source: PLoS One. 2024 Dec 31;19(12):e0309030. doi: 10.1371/journal.pone.0309030 (PMC11687914; doi:10.1371/journal.pone.0309030)
Supplement: S11 Table — (DOCX) [file pone.0309030.s011.docx]

|  | **Females (n = 154)** | **Males (n = 108)** |
| --- | --- | --- |
| **Age** | Mean = 31.1 years, range = 18-65 | Mean = 35.3 years, range = 18-67 |
| **Education** | Median = 3, range = 1-5 | Median = 2, range = 1-5 |

**S11 Table. Demographic variables grouped by sex**.

Sex but not gender was not assessed. Education levels: 1 = primary school, 2 = middle school, 3 = higher school, 4 = bachelor’s degree, 5 = master’s degree or higher.
