# Supplementary material for: Measuring habituation to stimuli: The Italian version of the Sensory Habituation Questionnaire
Source: PLoS One. 2024 Dec 31;19(12):e0309030. doi: 10.1371/journal.pone.0309030 (PMC11687914; doi:10.1371/journal.pone.0309030)
Supplement: S12 Table — (DOCX) [file pone.0309030.s012.docx]

|  | **Females (n = 154)** | **Males (n = 108)** |
| --- | --- | --- |
| **S-Hab-Q** | Mean = 25.77, range = 6-63 | Mean = 22.60, range = 5-49 |
| **SPQ** | Mean = 58.45, range = 24-94 | Mean = 56.54, range = 35-78 |
| **AQ** | Mean = 17.43, range = 3-39 | Mean = 19.12, range = 8-35 |

**S12 Table.** **Descriptive statistics of the questionnaires’ total scores grouped by sex.**

S-Hab-Q, Sensory Habituation Questionnaire; SPQ, Sensory Perception Quotient; AQ, Autism Quotient.
