# Supplementary material for: Measuring habituation to stimuli: The Italian version of the Sensory Habituation Questionnaire
Source: PLoS One. 2024 Dec 31;19(12):e0309030. doi: 10.1371/journal.pone.0309030 (PMC11687914; doi:10.1371/journal.pone.0309030)
Supplement: S13 Table — In the first model, sex and age factors were entered as predictors of the S-Hab-Q score. The factor sex was entered as predictor in the models predicting the S-Hab-Q score from the SPQ score and the S-Hab-Q score from the AQ score. (DOCX) [file pone.0309030.s013.docx]

| **Model** | **B (SE)** | **t** | ***p*** | |
| --- | --- | --- | --- | --- |
| S-Hab-Q ~ Sex × Age | | | | |
| SexM | -7.08 (2.74) | -2.58 | | **.010** |
| Age | - .07 (.04) | -1.36 | | .175 |
| SexM × Age | 0.12 (.07) | 1.58 | | .115 |
| S-Hab-Q ~ Sex × SPQ | | | | |
| SexM | 11.94 (5.63) | 2.12 | | **.003** |
| SPQ | .36 (.05) | 6.74 | | **< .001** |
| SexM × SPQ | - .25 (.09) | -2.63 | | **.009** |
| S-Hab-Q ~ Sex × AQ | | | | |
| SexM | 2.69 (3.57) | .75 | | .45 |
| AQ | .73 (.10) | 6.86 | | **< .001** |
| SexM × AQ | - .37 (.18) | -2.00 | | **.045** |

**S13 Table.** **Regression models.** In the first model, sex and age factors were entered as predictors of the S-Hab-Q score. The factor sex was entered as predictor in the models predicting the S-Hab-Q score from the SPQ score and the S-Hab-Q score from the AQ score.

S-Hab-Q, Sensory Habituation Questionnaire; SPQ, Sensory Perception Quotient; AQ, Autism Quotient; SexM = males were the reference level.
