# Supplementary material for: Measuring habituation to stimuli: The Italian version of the Sensory Habituation Questionnaire
Source: PLoS One. 2024 Dec 31;19(12):e0309030. doi: 10.1371/journal.pone.0309030 (PMC11687914; doi:10.1371/journal.pone.0309030)
Supplement: S14 Table — The R2 values refer to the combination of SPQ and S-Hab-Q in explaining the dependent variable. (DOCX) [file pone.0309030.s014.docx]

**S14 Table.** **Mediation model in females.** The R^2^ values refer to the combination of SPQ and S-Hab-Q in explaining the dependent variable.

|  | **Coefficient** | **β (SE)** | **z** | ***p*** | **Lower CI** | **Upper CI** |
| --- | --- | --- | --- | --- | --- | --- |
| AQ social skill ~ S-Hab-Q | b | .45 (.09) | 4.97 | **< .001** | .26 | .61 |
| AQ social skill ~ SPQ | c | -.07 (.08) | .77 | .440 | -.11 | .23 |
| S-Hab-Q ~ SPQ | a | .48 (.06) | 8.10 | **< .001** | .35 | .58 |
| Indirect effect | ab | .21 (.05) | 3.83 | **< .001** | .11 | .32 |
| Total effect | ab + c | .28 (.09) | 3.04 | **.002** | .09 | .45 |
| R^2^ = .23 |  |  |  |  |  |  |
