# Supplementary material for: Measuring habituation to stimuli: The Italian version of the Sensory Habituation Questionnaire
Source: PLoS One. 2024 Dec 31;19(12):e0309030. doi: 10.1371/journal.pone.0309030 (PMC11687914; doi:10.1371/journal.pone.0309030)
Supplement: S15 Table — (DOCX) [file pone.0309030.s015.docx]

**S15 Table. Mediation model table in males.**

|  | **Coefficient** | **β (SE)** | **z** | ***p*** | **Lower CI** | **Upper CI** |
| --- | --- | --- | --- | --- | --- | --- |
| AQ social skill ~ S-Hab-Q | b | .23 (.08) | 2.67 | **.008** | .07 | .39 |
| AQ social skill ~ SPQ | c | .06 (.07) | .79 | .426 | -.09 | .21 |
| S-Hab-Q ~ SPQ | a | .14 (.11) | 1.24 | .216 | -.08 | .35 |
| Indirect effect | ab | .03 (.03) | 1.25 | .261 | -.02 | .10 |
| Total effect | ab + c | .09 (.08) | 1.16 | .245 | -.06 | .24 |
| R^2^ = .06 |  |  |  |  |  |  |
