# Supplementary material for: Measuring habituation to stimuli: The Italian version of the Sensory Habituation Questionnaire
Source: PLoS One. 2024 Dec 31;19(12):e0309030. doi: 10.1371/journal.pone.0309030 (PMC11687914; doi:10.1371/journal.pone.0309030)
Supplement: S1 Fig — Probability density function (left panel) and quantile-quantile plot (q-q plot, right panel) of the questionnaires’ total scores. (DOCX) [file pone.0309030.s016.docx]

**S1 Fig.** **Distribution of the questionnaires’ scores.** Probability density function (left panel) and quantile-quantile plot (q-q plot, right panel) of the questionnaires’ total scores.


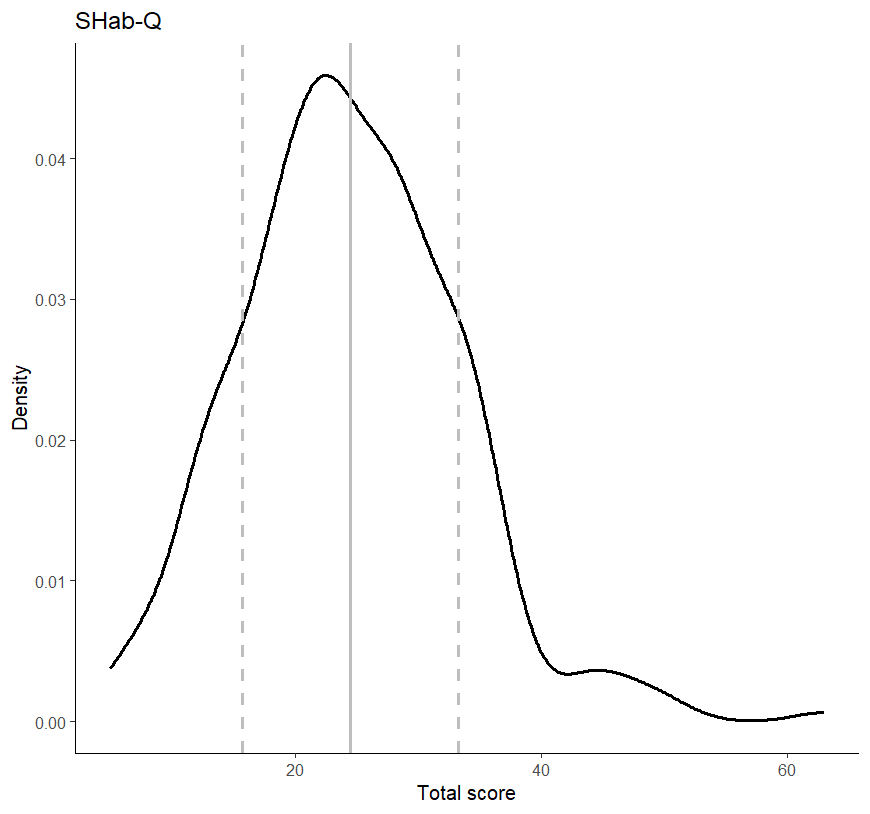

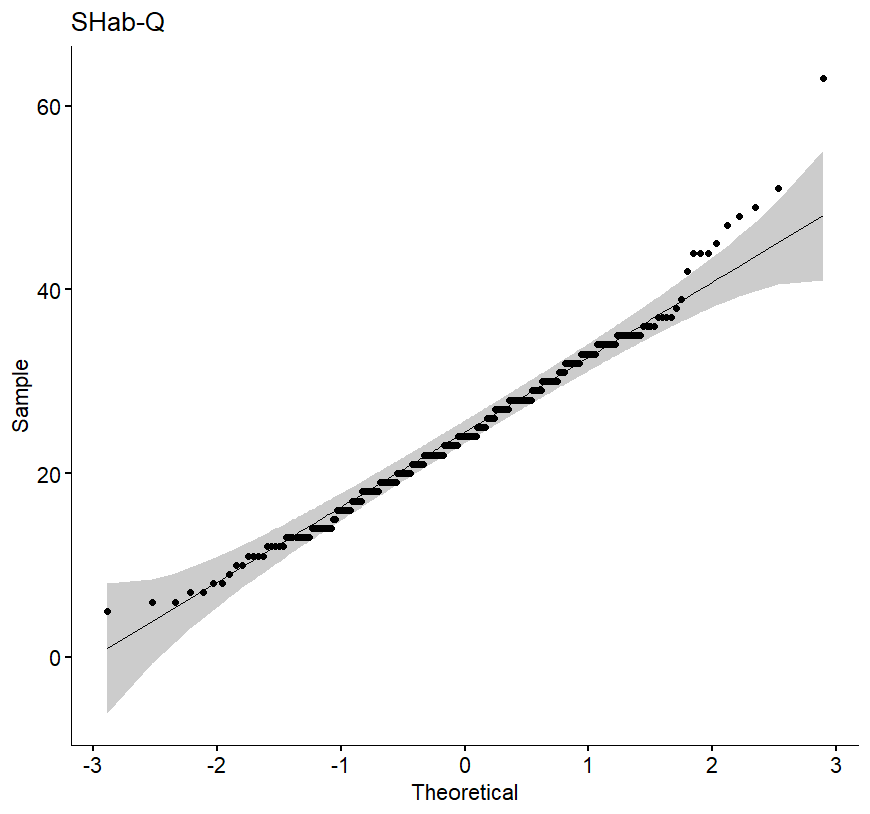


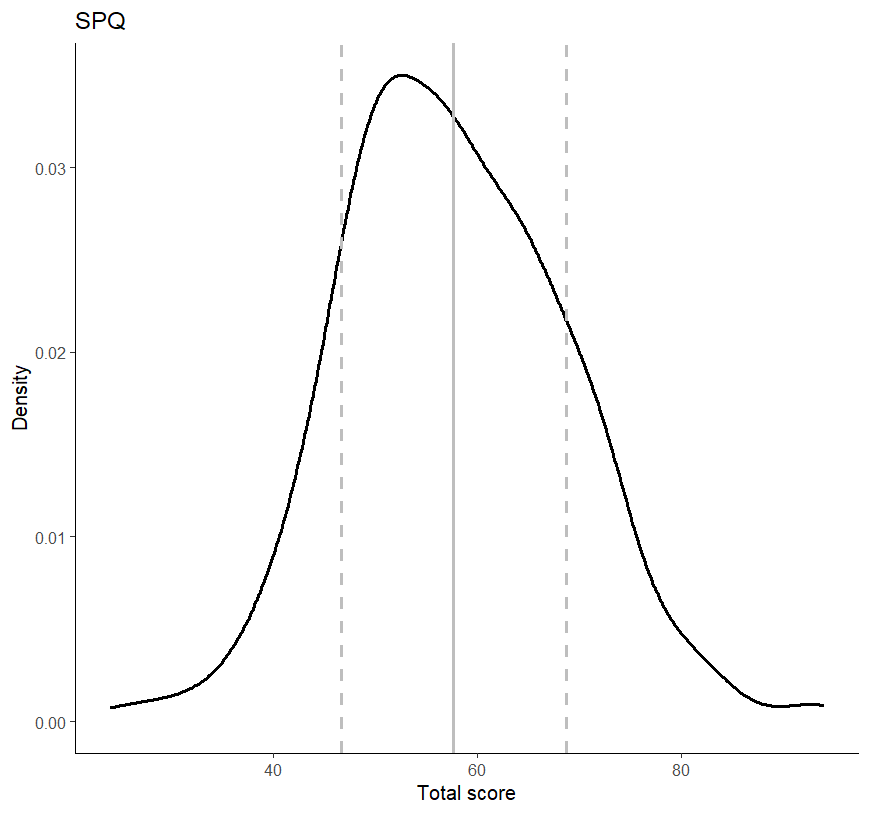

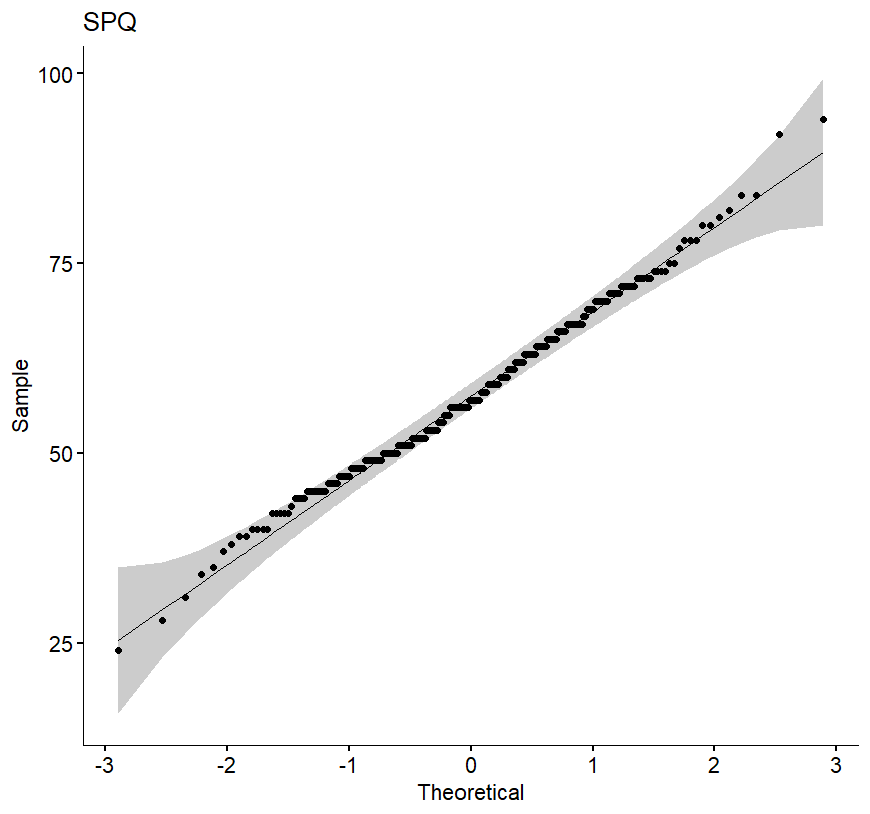


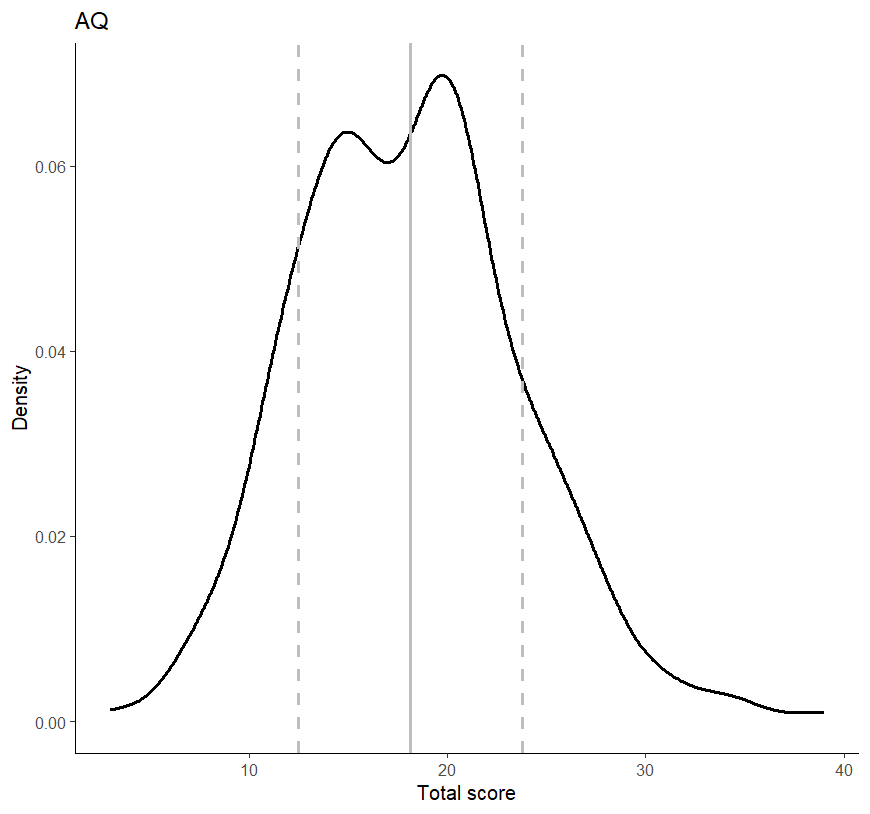

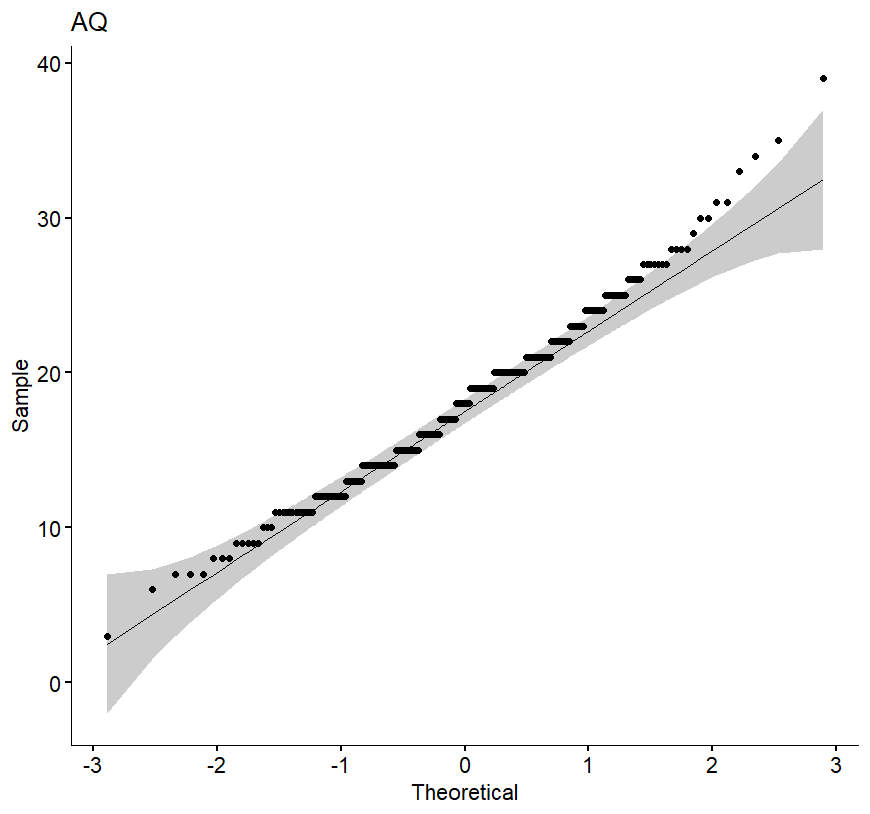


S-Hab-Q, Sensory Habituation Questionnaire; SPQ, Sensory Perception Quotient; AQ, Autism Quotient.
