# Supplementary material for: Measuring habituation to stimuli: The Italian version of the Sensory Habituation Questionnaire
Source: PLoS One. 2024 Dec 31;19(12):e0309030. doi: 10.1371/journal.pone.0309030 (PMC11687914; doi:10.1371/journal.pone.0309030)
Supplement: S2 Fig — (DOCX) [file pone.0309030.s017.docx]

**S2 Fig. Path diagram of the four-factor CFA analysis.**

.22

Q4

.5

Q13

.89

Q14

.47

Q2

.38

Q8

.47

Q1

.42

Q11

.87

Q15

.6

Q18

.46

Q22

.2

Q3

.53

Q5

.42

Q6

.67

Q7

.77

Q19

.35

Q10

.58

Q16

.48

Q24

.47

Q12

.3

Q21

.63

Q9

.49

Q17

.44

.26

.3

.3

.41

.37

.36

.5

.34

.35

.34

.34

.37

.43

.3

.32

.34

.3

.33

.39

Q23

.51

Q20

.48

.38

.17

.34

.37
