# Supplementary material for: Measuring habituation to stimuli: The Italian version of the Sensory Habituation Questionnaire
Source: PLoS One. 2024 Dec 31;19(12):e0309030. doi: 10.1371/journal.pone.0309030 (PMC11687914; doi:10.1371/journal.pone.0309030)
Supplement: S3 Fig — The analysis considered two models: AQ ~ S-Hab-Q + SPQ and S-Hab-Q ~ SPQ. The coefficient c represents the total effect of SPQ on AQ; c’ represents the direct effect of SPQ on AQ partialling out the effect of S-Hab-Q (c’ = c–ab). The mediation effect is ab and represents the indirect effect of SPQ on AQ through S-Hab-Q. (DOCX) [file pone.0309030.s018.docx]

**S3 Fig. Mediation model diagram for the AQ social skill subscale.** The analysis considered two models: AQ ~ S-Hab-Q + SPQ and S-Hab-Q ~ SPQ. The coefficient *c* represents the total effect of SPQ on AQ; *c’* represents the direct effect of SPQ on AQ partialling out the effect of S-Hab-Q (c’ = c – ab). The mediation effect is *ab* and represents the indirect effect of SPQ on AQ through S-Hab-Q.

SPQ

AQ

social skill

SHab-Q

c’ = -.14

b = .31

a = .37

1

.91

.86

c = - .03
