# Supplementary material for: Measuring habituation to stimuli: The Italian version of the Sensory Habituation Questionnaire
Source: PLoS One. 2024 Dec 31;19(12):e0309030. doi: 10.1371/journal.pone.0309030 (PMC11687914; doi:10.1371/journal.pone.0309030)
Supplement: S4 Fig — (DOCX) [file pone.0309030.s019.docx]

**S4 Fig. Mediation model for the attention switching AQ subscale.**

SPQ

AQ

attention switching

SHab-Q

c’ = -.01

b = .35

a = .37

1

.88

.86

c = .12
