# Supplementary material for: Measuring habituation to stimuli: The Italian version of the Sensory Habituation Questionnaire
Source: PLoS One. 2024 Dec 31;19(12):e0309030. doi: 10.1371/journal.pone.0309030 (PMC11687914; doi:10.1371/journal.pone.0309030)
Supplement: S5 Fig — (DOCX) [file pone.0309030.s020.docx]

**S5 Fig. Mediation model for the attention to detail AQ subscale.**

SPQ

AQ

attention to detail

SHab-Q

c’ = .39

9

b = -.08

a = .37

1

.86

.86

c = .36
