# Supplementary material for: Measuring habituation to stimuli: The Italian version of the Sensory Habituation Questionnaire
Source: PLoS One. 2024 Dec 31;19(12):e0309030. doi: 10.1371/journal.pone.0309030 (PMC11687914; doi:10.1371/journal.pone.0309030)
Supplement: S7 Fig — (DOCX) [file pone.0309030.s022.docx]

**S7 Fig. Mediation model for the imagination AQ subscale.**

SPQ

AQ

imagination

SHab-Q

c’ = -.06

9

b = .09

a = .37

1

.99

.86

c = -.02
