# Supplementary figures and images for: Measuring habituation to stimuli: The Italian version of the Sensory Habituation Questionnaire
Source: PLoS One. 2024 Dec 31;19(12):e0309030. doi: 10.1371/journal.pone.0309030 (PMC11687914; doi:10.1371/journal.pone.0309030)

**S8 Fig. Relationship between questionnaires’ scores grouped by sex.**

**
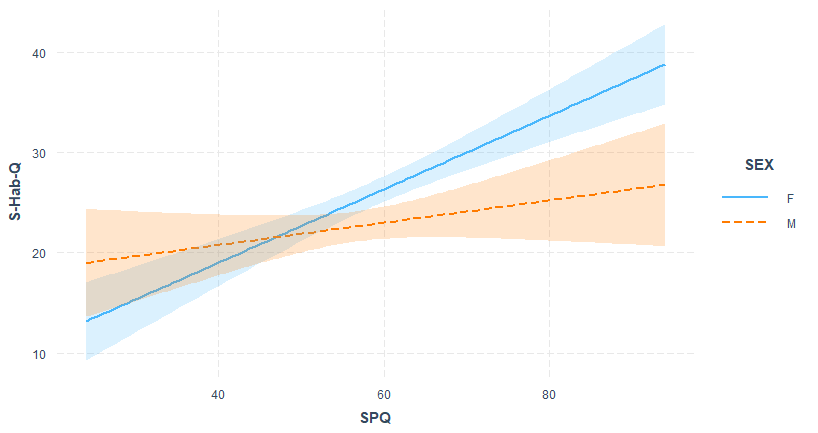
**

**
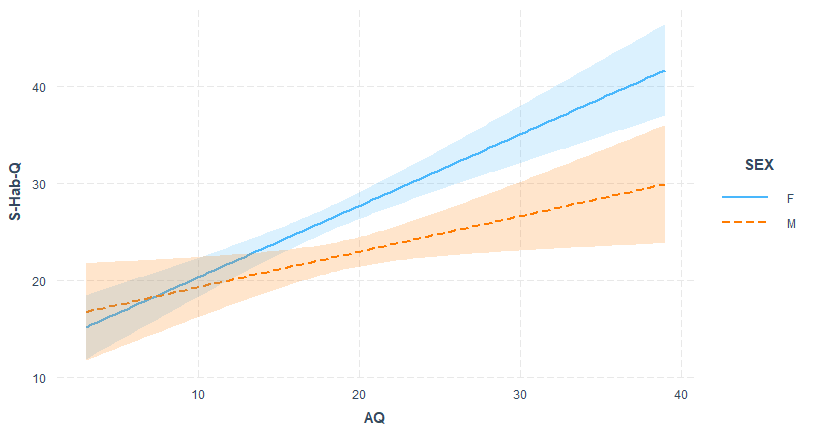
**

Supplement: S8 Fig — (DOCX) [file pone.0309030.s023.docx]
