# Supplementary material for: Measuring habituation to stimuli: The Italian version of the Sensory Habituation Questionnaire
Source: PLoS One. 2024 Dec 31;19(12):e0309030. doi: 10.1371/journal.pone.0309030 (PMC11687914; doi:10.1371/journal.pone.0309030)
Supplement: S10 Fig — (DOCX) [file pone.0309030.s025.docx]

**S10 Fig. Mediation model diagram in males.**

SPQ

SHab-Q

c’ = .06

b = .23

a = .14

1

.94

.98

c = .09

AQ
